# Perinatal derivatives: How to best characterize their multimodal functions *in vitro*. Part C: Inflammation, angiogenesis, and wound healing

**DOI:** 10.3389/fbioe.2022.965006

**Published:** 2022-08-04

**Authors:** Ana I. Flores, Caterina Pipino, Urška Dragin Jerman, Sergio Liarte, Florelle Gindraux, Mateja Erdani Kreft, Francisco J. Nicolas, Assunta Pandolfi, Larisa Tratnjek, Bernd Giebel, Michela Pozzobon, Antonietta R. Silini, Ornella Parolini, Günther Eissner, Ingrid Lang-Olip

**Affiliations:** ^1^ Regenerative Medicine Group, Research Institute Hospital 12 de Octubre (imas12), Madrid, Spain; ^2^ Center for Advanced Studies and Technology (CAST), Department of Medical, Oral and Biotechnological Sciences, University G. d’Annunzio Chieti-Pescara, StemTech Group, Chieti, Italy; ^3^ University of Ljubljana, Faculty of Medicine, Institute of Cell Biology, Ljubljana, Slovenia; ^4^ Laboratorio de Regeneración, Oncología Molecular y TGF-β, IMIB-Arrixaca, Murcia, Spain; ^5^ Service de Chirurgie Maxillo-Faciale, Stomatologie et Odontologie Hospitalière, CHU Besançon, Besançon, France; ^6^ Laboratoire de Nanomédecine, Imagerie, Thérapeutique EA 466, Université Bourgogne Franche-Comté, Besançon, France; ^7^ Institute for Transfusion Medicine, University Hospital Essen, University of Duisburg-Essen, Essen, Germany; ^8^ Department of Women’s and Children’s Health, University of Padova, Padova, Italy and Foundation Institute of Pediatric Research Fondazione Città Della Speranza, Padova, Italy; ^9^ Centro di Ricerca E. Menni, Brescia, Italy; ^10^ Department of Life Science and Public Health, Università Cattolica del Sacro Cuore, Rome, Italy; ^11^ Fondazione Policlinico Universitario “Agostino Gemelli” IRCCS, Rome, Italy; ^12^ Systems Biology Ireland, School of Medicine, Conway Institute, University College Dublin, Dublin, Ireland; ^13^ Division of Cell Biology, Histology and Embryology, Gottfried Schatz Research Center, Medical University of Graz, Graz, Austria

**Keywords:** perinatal derivatives, mesenchymal stromal cells, amniotic epithelial cells, amniotic membrane, functional assays, inflammation, angiogenesis, wound healing

## Abstract

Perinatal derivatives (PnD) are birth-associated tissues, such as placenta, umbilical cord, amniotic and chorionic membrane, and thereof-derived cells as well as secretomes. PnD play an increasing therapeutic role with beneficial effects on the treatment of various diseases. The aim of this review is to elucidate the modes of action of non-hematopoietic PnD on inflammation, angiogenesis and wound healing. We describe the source and type of PnD with a special focus on their effects on inflammation and immune response, on vascular function as well as on cutaneous and oral wound healing, which is a complex process that comprises hemostasis, inflammation, proliferation (including epithelialization, angiogenesis), and remodeling. We further evaluate the different *in vitro* assays currently used for assessing selected functional and therapeutic PnD properties. This review is a joint effort from the COST SPRINT Action (CA17116) with the intention to promote PnD into the clinics. It is part of a quadrinomial series on functional assays for validation of PnD, spanning biological functions, such as immunomodulation, anti-microbial/anti-cancer activities, anti-inflammation, wound healing, angiogenesis, and regeneration.

## 1 Introduction

Stem and progenitor cells from various tissues are increasingly being used in regenerative medicine and immunotherapies. For the expansion of these strategies, many researchers are putting effort on developing methods and technologies based on perinatal derivatives (PnD), which comprise perinatal tissues and thereof derived cells and secretomes. Besides perinatal mesenchymal stromal cells (MSC), also other perinatal tissues and cells, such as human amniotic membrane epithelial cells (hAEC), human amniotic fluid cells (hAFC), human parietal decidua (hPD) cells, or processed membranes (human amniotic membrane (hAM) or human amnio-chorionic membrane (hACM)) were already used or are under investigation for therapeutical treatment. Perinatal endothelial cells (EC) are predominantly isolated from the human umbilical vein (HUVEC), due to easier obtention and higher yield (Silini et al., 2020). Consequently, research using HUVEC is gaining momentum in the PnD field for the development of technologies useful for the vascular component regeneration and as an *in vitro* platform to validate the functional role of other PnD in the context of inflammation and angiogenesis (Pipino et al., 2022).

PnD portrait important advantages over products obtained from adults regarding their naïvity, availability and accessibility. This review aims to address the most widely studied PnD with a special focus on their functional *in vitro* validation in the field of inflammation, angiogenesis and cutaneous as well as oral wound healing to support their use in specific pre-clinical and clinical settings.

The anti-inflammatory properties of PnD make them highly attractive for treating inflammatory, autoimmune, and degenerative diseases (Silini et al., 2013; Cargnoni et al., 2021; Yang et al., 2021). Inflammation is the body’s defense mechanism to harmful stimuli, such as pathogens or damaged tissues. When the immune system is activated, inflammatory cells transmigrate from the vessels into damaged tissues (Medzhitov, 2008). There are two types of inflammation, acute inflammation characterized by a rapid response and chronic inflammation, which is a persistent but slowly evolving response. Immune cells from both, innate and adaptive response, play important roles in the pathogenesis of inflammatory diseases (Marshall et al., 2018). Chemokines as produced by damaged tissue recruit different immune cells, including eosinophils, macrophages, neutrophils, and T lymphocytes, contributing to inflammation. The resolution of inflammation is carried out by acting at different levels, such as through a decrease in the proliferation and maturation of immune cells, an increase in phagocytosis and apoptosis of immune cells, and an inhibition of the secretion of proinflammatory mediators. To behave as an anti-inflammatory agent, PnD should sense the inflammatory conditions, express and secrete anti-inflammatory molecules, and interact with immune cells. Accumulating evidence from preclinical and clinical trials indicates that PnD exert anti-inflammatory therapeutic effects in numerous autoimmune and inflammatory diseases such as graft versus host disease (GVHD), rheumatoid arthritis, multiple sclerosis, systemic lupus erythematosus, and respiratory diseases (Ringden et al., 2018; Yang et al., 2021; Ma et al., 2022).

Inflammation and angiogenesis, the formation of new blood vessels, are critical steps in the complex process of wound healing. Hemostasis, the first phase of wound healing, leads to vasoconstriction and the formation of a blot clot that stops bleeding. The following inflammatory phase leads to the accumulation of neutrophils and macrophages at the wound to defend bacteria and remove foreign substances, respectively. In addition, the adaptive immune system is activated. In the subsequent proliferative phase, fibroblasts multiply and deposit extracellular matrix, and angiogenesis, a critical component of acute wound healing, occurs. Re-epithelialization takes place as epithelial cells migrate from the periphery to the center of the wound. Further extracellular matrix deposition leads to a transition of the inflammatory state to a growth state, and a cross-linking of collagen, as well as scar maturation, finally leads to remodeling (Diegelmann and Evans, 2004; Dash et al., 2018). Similar processes have also been described in mucosal wound healing. Delayed wound healing is often due to insufficient blood supply based on impaired wound revascularization (Demidova-Rice et al., 2012). Further, chronic, non-healing wounds are detained in a self-perpetuating inflammatory stage that hinders progression to proliferation (Stojadinovic et al., 2008). Thus, chronic wounds are still a challenge to treat, although already more than a hundred years ago the hAM was used as a biological dressing for treating burns and skin ulcerations. Since then, while its use for the treatment of e.g. ocular ulcers is popular worldwide, its suitability for the management of skin ulcers is less well-recognized (Castellanos et al., 2017).

Improved techniques for tissue preservation, as well as advances in isolation and culture procedures for PnD cells facilitate the way for early phase clinical trials in diverse indications, such as the application of PLacental-eXpanded (PLX-PAD) stromal cells for the muscle recovery after hip arthroplasty (Winkler et al., 2022), multiple sclerosis (Lublin et al., 2014), pulmonary fibrosis (Chambers et al., 2014), and also COVID-19 (Hashemian et al., 2021; Sadeghi et al., 2021).

The focus of this review lies in the functional *in vitro* testing of PnD, which should be carried out before their application in animal models and subsequent clinical studies, to produce a treatment which is safe, effective and available for patients.

## 2 Mechanism of action

### 2.1 PnD effects on inflammation and the immune response

PnD can downregulate inflammation by acting on several key players in innate and adaptative immunity. PnD reduce inflammatory conditions *in vitro* by suppressing the proliferation, secretion of inflammatory cytokines, and cytotoxic activity of different immune cell subpopulations, as well as by inducing T cells and monocytes to acquire anti-inflammatory functions. These anti-inflammatory effects can be measured *in vitro* in cell-to-cell contact studies between PnD and immune cells (Erkers et al., 2013; Vellasamy et al., 2013), in non-contact transwell studies via secretion of soluble factors or by using conditioned medium (CM) of PnD cultures (Magatti et al., 2008; Gu et al., 2013). It is also important to understand that PnD can act constitutively (Magatti et al., 2008; Rossi et al., 2012; Pianta et al., 2015), and they can react to local inflammatory stimuli. To functionally assess this, an *in vitro* inflammatory environment created by the addition of Interferon γ (IFNγ) and/or Tumour Necrosis Factor (TNF), Interleukin (IL)1β, IL6, granulocyte-macrophage- colony stimulating factor (GM-CSF) or a mixture of all of these can be used to see if the cell morphology, size, immunophenotype and proliferation capabilities of PnD are altered (Mebarki et al., 2021). Furthermore, this simulated pro-inflammatory environment can be used to study the inhibitory capacity of PnD on T cell proliferation (Mebarki et al., 2021).

#### 2.1.1 Assessment of PnD effects on the innate immune response

Innate immune cells, such as macrophages, neutrophils, dendritic cells, and natural killer cells can be regulated by PnD (Abumaree et al., 2017; De La Torre et al., 2018; Edinger et al., 2018; Torre and Flores, 2020). Anti-inflammatory assays are focused on the evaluation of cell viability, maturation and/or activation of immune cells.

Macrophages play an important role in the initiation, preservation, and cessation of inflammation through production of several cytokines and growth factors. Generally based on their cytokine profile and cell surface markers (see below) macrophages show two different phenotypes: the predominantly pro-inflammatory M1 phenotype and the generally anti-inflammatory M2 phenotype The macrophage activity switches from pro-inflammatory (M1) to anti-inflammatory (M2) during the inflammatory process, and an imbalance of this change at the end of an inflammatory process is associated with a range of inflammatory diseases (Fujiwara and Kobayashi, 2005; Lee, 2018). Some studies have reported that PnD can induce anti-inflammatory effects by regulating macrophage functions. To study whether direct or indirect contact of PnD with macrophages induces a switch to an M2-like anti-inflammatory phenotype, a transwell chamber membrane culture system can be used. The effects of adding CM of unstimulated PnD to differentiated monocytes can also be evaluated. Firstly, CD14^+^ monocytes are isolated from peripheral blood mononuclear cells (PBMC) and differentiated into M1 macrophages by the addition of GM-CSF or lipopolysaccharide (LPS). Secondly, macrophages and PnD are co-cultured in the transwell chamber system and the M2 phenotype can be characterized by flow cytometric analyses of morphological changes and the expression of M2 cell surface markers (CD14, CD36, CD86, CD163, CD204, CD206, B7-H4 and CD11 b), the co-stimulatory molecules (CD40, CD80 and CD86) and the co-inhibitory molecules (CD273, CD274 and B7-H4), and major histocompatibility complex (MHC-II) molecules (Abumaree et al., 2013; Huang et al., 2019). The anti-inflammatory phenotype in M2-like macrophages can also be evaluated by qRT-PCR looking for an increased expression of the M2 cell surface markers, and by Enzyme-Linked Immunosorbent Assay (ELISA) examining increased secretion of IL-10 and arginase (Arg)-1, and decreased secretion of IL-1β, IL-12 (p70) and MIP-1α, TNF, inducible nitric oxide synthase (iNOS), and IL-6 (Abumaree et al., 2013; Huang et al., 2019). Furthermore, since the clearance of apoptotic cells is critical for the resolution of inflammation, it is possible to assess whether PnD increase the phagocytic activity of M2 macrophages. Phagocytosis of apoptotic cells is then evaluated by fluorescence microscopy (Abumaree et al., 2013) or the ability of M2 macrophages to take up zymosan particles as measured with the CytoSelect™ Phagocytosis Kit (Abumaree et al., 2013). The immortalized human monocyte/macrophage cell line THP-1 is another useful cell model system to study the effect of PnD on activated macrophages (He et al., 2012). THP-1 cells are pre-treated with phorbol-12-myristate-13-acetate (PMA) to induce macrophage differentiation and then activated with LPS (Shu et al., 2015). After direct coculture with PnD or their CM, Western blot can be used to detect diminished levels of the proinflammatory cytokines TNF and IL-1β, and the inhibition of the mitogen-activated protein kinase (MAPK)/NF-κB signaling pathway (Hu et al., 2013). Macrophages can induce changes in the phenotype of PnD, and these changes can be evaluated by flow cytometry as an induction in secretion of the inflammatory proteins IL8, IL-12, and Monocyte Chemoattractant Protein 1 (MCP-1), and of anti-inflammatory proteins IL-10, indoleamine-pyrrole-2,3-oxygenase (IDO) and B7H4 (Abumaree et al., 2013).

Neutrophils along with macrophages provide the innate cell-mediated immunity and inflammatory responses at sites of injury. Indeed, cytokines secreted by macrophages attract neutrophils to the injured area to initiate the inflammatory response that in turn will recruit additional innate immunity cells and molecules. To assess the role of PnD in neutrophil and macrophage migration *in vitro*, their chemotactic activity towards recombinant macrophage inflammatory protein (MIP)-2 could be tested in the presence of CM of PnD using migration assay chambers (Li et al., 2005). To study whether PnD induce neutrophil-like N2-type polarization in an inflammatory environment, a trans-well system is used under stimulation of neutrophils with pro-inflammatory LPS. In these *in vitro* systems, two different cell models of neutrophils can be used, neutrophils freshly isolated from human blood, and the human leukemia cell line (HL-60) that is an incomplete differentiated cell line that must undergo differentiation to become functional neutrophils (Babatunde et al., 2021), HL-60 cells differentiate into neutrophils with DMSO, and subsequent stimulation with LPS induces a polarized N1 phenotype. qPCR and Western blot are used to determine the neutrophil surface molecules associated with a polarized N2-phenotype as well as the levels of secreted pro-inflammatory and anti-inflammatory factors (Wang et al., 2020). Furthermore, PnD also have direct effects on major functions of neutrophils. Co-culture of freshly isolated human blood neutrophils with PnD can serve to demonstrate the increased phagocytic capacity of neutrophils and, at the same time, their decreased oxidative burst capacity as another method to validate the anti-inflammatory function of PnD (Magatti et al., 2018; Alipour et al., 2020). However, there are not many studies addressing the anti-inflammatory role of PnD on neutrophils despite the increasing evidence revealing unsuspected roles of neutrophils in many physiological and pathological processes (Nicolas-Avila et al., 2017), and more studies are needed.

As part of the anti-inflammatory effects of PnD, the inhibition of the differentiation and maturation of monocytes to dendritic cells (DC) has been reported. Monocytes isolated from peripheral blood are differentiated to immature dendritic cells (iDC) in the presence of IL-4 and GM-CSF, and iDCs develop into mature dendritic cells (mDC) in the presence of LPS. Both processes are inhibited by PnD, by direct or indirect contact with the DC using a transwell culture system, or by the addition of CM of PnD (Magatti et al., 2009; Croxatto et al., 2014; Abomaray et al., 2015). Furthermore, PnD induce the switch to an anti-inflammatory phenotype in both iDC and mDC. This change can be measured by flow cytometry, observing a decrease in the expression of co-stimulatory molecules (CD40, CD80, CD83 and CD86) and an increase in the expression of co-inhibitory molecules (B7H3, B7H4, CD273, CD274), and of the immunosuppressive enzyme IDO (Abomaray et al., 2015). Likewise, PnD block the production of pro-inflammatory cytokines by iDC and mDC such as TNF, IL-6, IL-12 and IL-23, IFNγ, C-X-C motif chemokine ligand 10 (CXCL10), CXCL9 and chemokine C-C motif ligand (CCL5) while the secretion of IL-10 is increased, as determined in cell culture supernatants using the ELISA assay (Magatti et al., 2009; Abomaray et al., 2015; Magatti et al., 2015). Phagocytic activity of iDC is essential for the elimination of the cellular components released after a tissue injury, which -if accumulated-would cause inflammation. Phagocytic activity of iDC is induced by PnD and can be determined by CytoSelect™ phagocytosis functional assay (Abomaray et al., 2015). DC are antigen presenting cells capable of inducing an efficient T cell response to specific antigens, being an important mediator of the innate and adaptive immune response. To directly determine the impact of DC co-cultured with PnD on T-cell proliferation, a mixed lymphocyte reaction (MLR) is used, and T cell proliferation can be quantified by ^3^H thymidine uptake (Croxatto et al., 2014; Magatti et al., 2015; Talwadekar et al., 2015). Then, PnD induce an anti-inflammatory phenotype on DC which will down-regulate the activity and proliferation of T cells, presenting *in vitro* evidence of their role in modulating the immune response in immunological diseases.

In most of these studies, monocytes are isolated from human PBMC, and only few of them used an established cell line model. Although it is well known that the composition of human PBMC depend on the donor’s physiological status and its use supposes a high variability across donors (Kleiveland, 2015), they are the gold standard tool for isolating monocytes to investigate the role of immune cells in inflammatory diseases. However, although monocyte-like cells do not fully replicate the genotypic and phenotypic properties of human peripheral blood monocytes (Riddy et al., 2018), they represent a simplified surrogate and readily available cell model, especially when human blood is not available, which makes them a valuable and convenient model for repeated testing.

Natural killer cells (NK cells) are large granular lymphocytes with cytotoxic activity against injured cells, and their activation results in the secretion of pro-inflammatory cytokines, suggesting that NK cells can either drive inflammation or restrain adaptive immune responses to prevent excessive inflammation or even autoimmunity (Brandstadter and Yang, 2011; Zitti and Bryceson, 2018; Moloudizargari et al., 2021). Analysis of cellular crosstalk between PnD and NK cells has yielded contradictory results probably due to either the different origin of PnD and the different microenvironmental conditions to which the cells are exposed during pregnancy (Abumaree et al., 2019) or due to different PnD:NK and NK/tumor cells ratios used in each study (Moloudizargari et al., 2021), or to the different cell type used for cytotoxicity testing (Boissel et al., 2008). Despite this, it is valuable to know how PnD interact with NK cells in culture. It is possible to determine whether the coculture modulates NK cell proliferation, NK-activated receptor expression (NKp30, NKp44, NKp46, NKG2D, and CD69) and/or NK cell cytotoxicity on tumor cell lines (Chatterjee et al., 2014; Croxatto et al., 2014; Li et al., 2015). Furthermore, the profile of cytokine production by NK cells may be affected by coculture with PnD resulting in changes in the expression of pro-inflammatory molecules that can be measured by RT-PCR and/or ELISA assay (Li et al., 2015; Abumaree et al., 2019). Furthermore, the suppressive activity of PnD can be measured by their release of anti-inflammatory molecules such as IL-10 and prostaglandin E2 (PGE2) when cocultured with NK cells (Chatterjee et al., 2014; Li et al., 2015). Interestingly, IL-2 preactivated NK cells exert cytotoxic effects on PnD despite expressing high levels of HLA-I, whereas nonactivated NK cells did not lyse the PnD, suggesting that an inflammatory environment is necessary for the NK cytolytic activity against PnD (Abumaree et al., 2018; Abumaree et al., 2019).

#### 2.1.2 Assessment of PnD effects on the adaptative immune response

PnD have anti-inflammatory properties acting on the adaptive response and these are measured as their ability to inhibit the proliferation and cytokine production of T lymphocytes as well as their ability to modulate T cell differentiation. The anti-inflammatory properties of PnD can be studied using *in vitro* models of inflammatory diseases, such as ocular allergic inflammation (Solomon et al., 2005), inflammation of human middle ear epithelial cells (HMEEC) by airway pollutants (Kim et al., 2019; Kim et al., 2020), atopic dermatitis (Kim et al., 2020) or demyelinating diseases (Bravo et al., 2016). The anti-inflammatory effects of PnD after co-culture can be analyzed by fibroblast proliferation measured with the [^3^H]-thymidine incorporation assay (Solomon et al., 2005) or T cell proliferation measured by the MTT assay (Bravo et al., 2016). The inhibitory effects can be analyzed by RT-PCR and ELISA showing the down-regulation of inflammatory cytokines released by fibroblasts, such Trasnforming Growth Factor (TGF)-β1, GM-CSF, IL-8, IL6, TNF, IL1β, and thymus as well as activation-regulated chemokines (TARC) (Solomon et al., 2005; Kim et al., 2019; Kim et al., 2020) or down-regulation of the inflammatory cytokine IL17 released by T cells (Bravo et al., 2016). In addition, the levels of PnD-expressed anti-inflammatory genes in these inflammatory systems, such as PGE2, TGFβ, and Vascular Endothelial Growth Factor (VEGF), can also be measured (Kim et al., 2019).

As described throughout this chapter, the anti-inflammatory potential of PnD is primarily assessed by determining self-secreted cytokines, as well as the production of pro- and anti-inflammatory cytokines by immune cells. ELISA and flow cytometry are the most widely used and best validated methods to measure cytokines and other inflammatory mediators. However, these methods are time-consuming, require a long sample preparation time, and do not allow the measurements of multiple cytokines at the same time in the same sample and in real-time. Recently, multiplex arrays (Luminex-based) have been developed from traditional ELISAs which allows the measurement of multiple cytokines in the same sample at the same time, and using only a small volume (Leng et al., 2008). Other commonly used immunoassays include Meso Scale Discovery (MSD), cytometric bead array (CBA), time-resolved fluorescence resonance energy transfer (TR-FRET), AlphaLISA, and FirePlex which have different sensitivities and multiplexing capabilities (Platchek et al., 2020). These technologies could represent more reliable and simpler strategies to assess the effect of PnD on immune cells.

### 2.2 PnD effects on inflammation and the vascular function

Among PnD, endothelial cells derived from the placenta (hP-EC) and umbilical cord, such as HUVEC represent a precious easy access *ex vivo* model of the human vasculature (Silini et al., 2020). HUVEC applications range from cardiovascular to metabolic as well as wound healing and angiogenesis-related diseases (Medina-Leyte et al., 2020). In addition, Gestational Diabetes (GD)-HUVEC exposed *in vivo* even transiently (during pregnancy) to hyperglycemia, exhibit some epigenetics modifications leading to a durable pro-inflammatory phenotype (Di Fulvio et al., 2014; Di Pietrantonio et al., 2021). HUVEC have been used to study the pro- and anti-atherogenic effect of several molecules in the early stages of atherosclerosis (Di Tomo et al., 2015; Di Pietro et al., 2020). Therefore, they represent a valuable *in vitro* model to assess the role of PnD in the above diseases through the following functional assays.

Due to a pro-oxidant state of Gestational Diabetes (GD)-HUVEC can be stimulated with an oxidative stress agent and compared to control HUVEC. O2^-^ production and intracellular accumulation of reactive oxygen species (ROS) are measured to assess the antioxidant effect of PnD. While these methods allow to assess the general oxidative status, more integrative approaches evaluating potential antioxidant effects with higher sensitivity as well as the identification of permanent markers of oxidative stress are required (Pipino et al., 2020). Therefore, the levels of DNA/RNA damage, lipid peroxidation, and protein oxidation/nitration such as nitrotyrosine expression associated with impaired vascular nitric oxide (NO) secretion and availability may give a more reliable scenario regarding alteration of the oxidative balance (Di Fulvio et al., 2014; Alshabibi et al., 2018; Di Pietrantonio et al., 2021).

In endothelial dysfunction, the early stage of atherogenesis, there is a close interaction between oxidative stress and inflammation. Increased monocyte adhesion to the endothelium is among the mechanisms predisposing to endothelial dysfunction, the early predictor of plaque formation and atherosclerosis. The Monocyte-HUVEC Adhesion Assay can be performed in C- and GD-HUVEC in the basal state and after incubation with PnD before stimulation with an inflammatory stimulus such as low doses of TNF. Subsequently, cells from the monocytic cell line U937 are added to evaluate their adhesion to HUVEC monolayers (Di Tomo et al., 2015). Moreover, by qPCR and western blot/flow cytometry any inflammation-related gene and protein, such as Vascular Cell Adhesion Molecule 1 (VCAM-1) and Intercellular Adhesion Molecule 1 (ICAM-1), can be evaluated. Indeed, it is noteworthy that the level of these adhesion molecules increases during inflammation. More importantly, VCAM-1 and ICAM-1 membrane exposure, the main mechanism behind the interaction between monocytes and endothelial cells, can be analyzed by flow cytometry (Di Tomo et al., 2015).

Furthermore, it is accepted that impaired NO synthesis and/or its availability in HUVEC may result in endothelial dysfunction (Pandolfi and De Filippis, 2007). To assess NO bioavailability, the intracellular cGMP level, a biological target of NO activity, is mainly evaluated by using a commercial enzyme immunoassay (EIA) kit (Di Pietro et al., 2020). It has been demonstrated that HUVEC chronically exposed to high glucose and inflammation, as well as treated with the proinflammatory stimulus TNF-α, display decreased levels of cGMP (Ucci et al., 2019) Therefore, this assay is useful to assess the role of PnD in changes of NO bioavailability, which is involved in the modulation of the Nuclear Factor kappa-light-chain-enhancer of activated B cells (NF-κB) nuclear translocation, relevant to further evaluate the potential ability of PnD in inhibiting the inflammatory pathway (Ucci et al., 2019). However, accurate NO detection and quantification are critical to understanding health and disease. Consequently, more than one of the aforementioned assays should be performed for a clear comprehension of anti-inflammatory PnD effects.

Although these assays are widely used for reproducible determinations, the results obtained may be partially affected by the passage number and donor variability of endothelial cells. To overcome these issues, experiments may be performed on multiple endothelial cell strains or immortalized endothelial cells, showing a more uniform response.

Moreover, in some inflammatory diseases, the release of several pro-inflammatory chemokines may inhibit neovascularization. The capability of PnD to regulate the chemokine environment by inhibiting the pro-inflammatory chemokines and/or by increasing the pro-angiogenic ones, may enhance new vessel formation. The most conventional way of assessing PnD’ capacity in decreasing inflammation while improving endothelial cell network-like structures is the Matrigel tube formation assay (Ma et al., 2021; Nensat et al., 2021). This assay, together with the evaluation of cell migration through a scratch assay (Bernabe-Garcia et al., 2017), can be performed to assess the potential therapeutic efficacy of PnD in angiogenesis-related disease, such as diabetic foot ulcers (Castellanos et al., 2017). Besides the rapid and easy well-established method and the comparably easy cell culture and measurement, this assay is only partially representative of real cell environments. Although many laboratories commonly use this method to obtain first evidence of angiogenic and antiangiogenic agents, some limitations may occur such as difficult in vessel quantification through a specific plugin of ImageJ software as well as standardization due to Matrigel lot-to-lot variability (Nowak-Sliwinska et al., 2018). The latter may be prevented by selecting a specific lot with preferred protein and endotoxin concentrations.

Overall, all the HUVEC assays mentioned here were recently published in a study performed to assess the anti-inflammatory and pro-angiogenic role of hAM in GD-HUVEC. The results obtained strongly elucidate the mechanisms through which hAM can affect inflammation, migration, and angiogenesis thus providing additional validation for ongoing clinical trials in diabetic foot ulcer (Pipino et al., 2022).

In addition, to overcome limitations of 2D cell culture assays, HUVEC can be used *in vitro* to create 3D spheroid structures for functional screening purposes (Kocherova et al., 2019) or blood vessel tissue engineering (Moby et al., 2007; Paternotte et al., 2013). Finally, endothelial cells and the anti-inflammatory influence of PnD can also be analyzed even more physiologically under shear stress conditions in dedicated flow chambers with a computer-guided pump system and video microscopy (Zantl and Horn, 2011; Munir et al., 2015). However, all these methods do not acknowledge the multicellularity in the perivascular microenvironment. An *in vitro* solution to this problem would be vascularized spheroids or organoids which are currently under investigation.

### 2.3 PnD effects on cutaneous wound healing

Substantial evidence from several clinical trials shows how the application of hAM or other PnD on wounds and ulcers of diverse etiology has proven to be of benefit (Colocho et al., 1974; Singh et al., 2004; Hasegawa et al., 2007; Mermet et al., 2007; Silini et al., 2015). Additionally, a combination of PnD or even extra-cellular vesicles from PnD have been used for chronic wounds (Bakhtyar et al., 2018; Hashemi et al., 2019). All that evidence makes hAM and derived products the most researched so far in terms of wound healing treatment. Nevertheless, to fully understand the capabilities of hAM and other PnD to promote wound healing, several *in vitro* systems have been developed. Most chronic wounds show decreased proliferation and migration, mainly affecting keratinocytes, but also fibroblasts and endothelial cells. In consequence, those experimental settings mainly focus on evaluating the pro-proliferative, pro-migratory and pro-angiogenic effects that hAM or other PnD may have.

**FIGURE 1:**
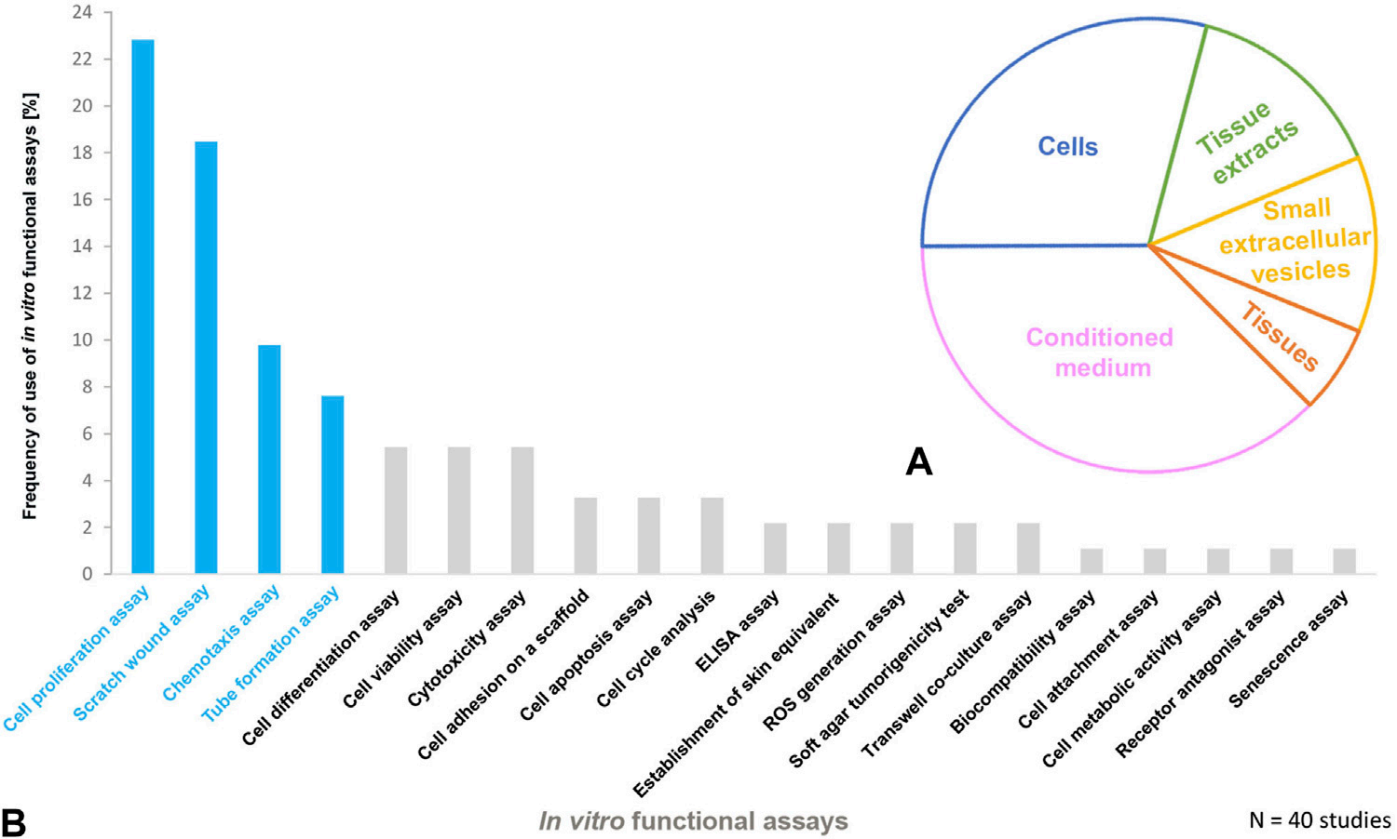
Perinatal derivatives and *in vitro* functional assays used for cutaneous wound healing in animals **(A)** The schematic presentation of PnD functionally tested *in vitro* and **(B)** frequency of use of *in vitro* functional assays in animal studies of cutaneous wound healing. The most frequently used functional assays are highlighted in blue.

In addition, *in vivo* wound healing assays using animal models constitute an integrative benchmark in which PnD′ effects on inflammation and the immune component, such as angiogenesis and dermal as well as epidermal cell migration and proliferation, have to cooperate to show real functional effectiveness. The application of human PnD in animal studies of cutaneous wound healing was extensively reviewed by Pichlsberger et al. (Pichlsberger et al., 2021). In approximately 50% of the included studies (Pichlsberger et al., 2021) the PnD were functionally tested *in vitro* before they were applied to animal wounds. Herein we focus on the *in vitro* functional assays used in these studies. The outcome of these *in vitro* assays, the types and combinations of the PnD applied as well as the references are outlined in detail in Tables 1–5 **.** As naming and abbreviations of the PnD types in the reviewed studies varied due to the authors’ discretion, we harmonized terms according to the recently published consensus nomenclature for PnD to improve the comparability of data (Silini et al., 2020). Among the included studies, the majority of the *in vitro* tested PnD were cells (38%) mainly MSC isolated from the umbilical cord, amnion, amniotic fluid, placenta, but also cells derived from the amniotic fluid, and the amniotic epithelium. Further functional tests were performed on cellular secretomes (29%), followed by cell-derived small extracellular vesicles (sEVs, 15%) derived from MSC of the umbilical cord or decidua, tissue extracts (13%) and tissue membranes (amnion or amnion/chorion, 6%) (Figure 1A). We did not discriminate between the different subtypes of extracellular vesicles (EV) including exosomes etc., but chose the term sEVs instead, according to the recommendations of the International Society for Extracellular Vesicles to use an operational term for EV subtypes unless their endosomal origin was proven (Van Deun et al., 2017; Thery et al., 2018). Overall, these studies used twenty different functional assays to evaluate PnD *in vitro* (Figure 1B). The most frequently performed functional assays were: 1) cell proliferation assay, 2) scratch wound assay, 3) chemotaxis assay and 4) angiogenesis assays. These assays are considered the gold standard for the evaluation of wound healing *in vitro* since they analyze important processes that occur during wound healing, such as proliferation and migration of the cells at the wound edges and the re-epithelialization of the wound surface.

**TABLE 1:** Functional tests on perinatal cells.

**Perinatal cells**			
**PnD Cell type**	**Functional *in vitro* tests**	**Outcome**	**Reference**
hAFSC	Establishment of skin equivalent	hAFSC differentiated into keratinocytes expressing K5, K14, K10, and involucrin after 30 days of culture in a keratinocyte-inducing medium and formed a complete pluristratified skin epithelium under air-liquid culture conditions on a collagen matrix with integrated HDF.	(Sun et al., 2015)
hAMSC	Cell adhesion on a scaffold	hAMSC were grown on Matriderm and PCL/PLA scaffolds. PCL/PLA yielded a higher number of attached cells and more favorable growing conditions for hAMSC than Matriderm.	(Vonbrunn et al., 2020)
hUC-MSC	(i) Cell proliferation assay	c-Jun silencing in hUC-MSC inhibited (i) cell proliferation and (ii) migration, while c-Jun overexpression enhanced proliferation but not migration of hUC-MSC.	(Yue et al., 2020)
	(ii) Scratch wound assay		
	Cell differentiation assay	hUC-MSC were transfected with a lentivirus expressing HOXA4 and cultured for 21 days. Expression of the epidermal cell-specific markers, cytokeratins 14 and 18, was detected by immunohistochemistry and flow cytometry.	(He et al., 2015)
	Establishment of skin equivalent	hUC-MSC were seeded on the surface of fibrin gel scaffolds and cultured for 7–10 days. The established equivalent resembled the normal skin architecture.	(Montanucci et al., 2017)
	(i) Cell proliferation assay	(i-iii) SAP improved the survival, proliferation, and migration of the hUC-MSC encapsulated in Pluronic F-127 hydrogel (drug delivery scaffold).	(Deng et al., 2020)
	(ii) Cell viability assay	
	(iii) Scratch wound assay	
	(i) Cell proliferation	(i, ii) Activation of the Wnt signaling pathway promoted survival of hUC-MSC (proliferation, viability) seeded on a CCLDADM scaffold. (iii) Cells attached and grew uniformly when seeded onto the CCLDADM scaffold.	(Han et al., 2019)
	(ii) Cell viability assay	
	(iii) Cell adhesion on a scaffold	
	(i) Cell proliferation assay	455-nm blue light exposure effectively promoted (i) proliferation, (ii) migration, and (iii) tube formation of HUVEC co-cultured with hUC-MSC.	(Yang et al., 2019)
	(ii) Scratch wound assay	
	(iii) Tube formation assay	
	Chemotaxis assay	hUC-MSC seeded in 3D alginate gel gradually migrated from the top to the bottom of the gel, but could not migrate out from the gel during 7 days of observation.	(Wang et al., 2016b)
	Soft agar tumorigenicity test	Even after repeated passaging the cells have not acquired tumor formation capabilities.	(Sabapathy et al., 2014)
a) hUC-MSC	(i) Cell proliferation assay	hUC-MSC-End showed increased (i) proliferation, (ii) migration and (iii) vasculogenesis compared to hUC-MSC.	(Kaushik and Das, 2019)
b) hU-MSC-End	(ii) Chemotaxis assay		
	(iii) CAM assay		
hPMSC	Transwell co-culture assay	Co-culturing of hPMSC with HDF inhibited LPS-induced activation of NF-ĸB signal in HDF.	(Wang et al., 2016a)
	Chemotaxis assay	hPMSC expressing PDGFR-β exhibited enhanced chemotactic migration compared to hPMSC without expressing PDGFR-β.	(Wang et al., 2018)
a) hAEC b) hUC-MSC	Cell differentiation assay	hAEC and hUC-MSC were able to differentiate into keratinocytes and fibroblasts, respectively, after 15 days of culturing in an inducing medium. This was shown by the expression of various specific markers by immunolabeling and RT-PCR.	(Mahmood et al., 2019)

Abbreviations: CCLDADM: collagen–chitosan laser drilling acellular dermal matrix, hAEC: human amniotic membrane epithelial cells, hAFSC: human amniotic fluid stem cells, hAMSC: human amniotic membrane mesenchymal stromal cells, HDF: human dermal fibroblasts, hPMSC: human placenta mesenchymal stromal cells, hUC-MSC: human umbilical cord mesenchymal stromal cells, hU-MSC-End: human umbilical cord mesenchymal stromal cells -endothelial transdifferentiated, HUVEC: human umbilical cord vein endothelial cells, LPS: lipopolysaccharides, NF-ĸB: nuclear factor kappa-light-chain-enhancer of activated B-cells, PCL/PLA: Poly(caprolactone)/poly(l-lactide), PDGFR-β: platelet derived growth factor receptor β, SAP: sodium ascorbyl phosphate.

From the pathological point of view, an important limiting factor for wound healing could be the development of an altered keratinocyte behavior (Raja et al., 2007). In that sense, many authors have measured the ability of amnion or other PnD in promoting keratinocyte migration in *vitro*. Next to the well-known Boyden chamber migration assay (Mcquilling et al., 2019), the wound healing scratch assay is frequently used. By design, based on measuring the gap generated by mechanically tearing a cultured cell monolayer, the scratch assay allows for easy monitoring and quantification of cell migration and wound closure (Liarte et al., 2018). The unique advantage of this assay is the fact that cells at the wound edge can be studied microscopically at any time, allowing for the characterization of distinct morphology and topological changes in living cultures, along with the precise detection of the expression of key factors involved in wound healing on fixed cultures (Bernabe-Garcia et al., 2017). Of note, the wound healing scratch assay can be applied on almost any cell line growing in monolayers. For instance, in the specific case of skin wounds, most of the preclinical experience with amnion and other PnD is based on the effects of well stablished human keratinocyte cell lines, such as the spontaneously transformed aneuploid immortal human cell line HaCaT (Boukamp et al., 1988). However, several reports also apply the scratch method onto human fibroblasts (Bakhtyar et al., 2018; Rahman et al., 2019). In the case of keratinocytes, HaCaT offers unparalleled characteristics by being phenotypically similar to primary keratinocytes, while still allowing routine culture procedures (Boukamp et al., 1988). Numerous papers show that HaCaT keratinocytes had not only allowed to certify the benefits of amnion on migration (Alcaraz et al., 2015; Kitala et al., 2019; Rahman et al., 2019) but also to understand how amnion treatment effects appear to be restricted to the edge of the artificial wound *in vitro* (Ruiz-Canada et al., 2018). These results support the notion that migration effects of amnion are not systemic, but local. This is in line with clinical observations that the effects of amnion treatment of human patients’ wounds were limited to the wound edge (Insausti et al., 2010). Moreover, the HaCaT scratch assay in combination with immunological methods allows for the precise detection of key proteins participating in the migration machinery, such as Paxilline H, demonstrating that among its effects, amnion treatment triggers focal adhesion molecule rearrangement in cells at the very edge of the wound and is thus promoting local cell migration (Bernabe-Garcia et al., 2017). Altogether, the possibilities provided by the wound healing scratch assay offer uncontested capacity to obtain high quality data in a comprehensive manner, opening opportunity for precise correlations between what is macroscopically observed in the chronic wounds and the behavior of cells observed *in vitro*, as shown for the HaCaT cells, (Ruiz-Canada et al., 2021).

A proper development of the underlying dermal tissue is a prerequisite for keratinocyte proliferation as an important limiting factor for correct healing and wound closure. Therefore, fibroblast and endothelial cell proliferation also needs to be assessed. Using different methodologies, from image analysis to comparative cell counting, including cell cycle analysis and cell suspension absorbance determination, numerous papers evaluate the effects of amnion treatment and coincide in finding positive effects on keratinocyte and fibroblast proliferation (Alcaraz et al., 2015; Murphy et al., 2017; Kitala et al., 2019; Rahman et al., 2019).

In contrast, comparably little is known about the effects of amnion or other PnD on endothelial cell proliferation. However, studies assessing this component mostly apply methods based on the analysis of *in vitro* tube formation and branching by endothelial cells in culture (Hu et al., 2018; Bullard et al., 2019). Tube formation assay, an *in vitro* angiogenesis assay, is often used in wound healing experiment designs since the formation of new vessels significantly contributes to tissue recovery. The 3D-tube formation assay on gelled basement membrane extract is a powerful *in vitro* technique for evaluating angiogenesis. This assay involves endothelial cell adhesion, migration, protease activity, and tube formation (Arnaoutova and Kleinman, 2010). Also, 2D co-culture models are meaningful assays to evaluate the functionality of the respective PnD type. For example, endothelial cells showed a significantly increased formation of vessel-like structures in direct co-culture with MSC derived from placental blood vessels as compared to human amniotic membrane mesenchymal stromal cells (hAMSC) (Konig et al., 2015). The angiogenic properties of PnD were also evaluated by the chorioallantoic membrane (CAM) assay (Edwards et al., 2014; Rameshbabu et al., 2018; Kaushik and Das, 2019). The CAM assay is an intermediate step between *in vitro* and the *in vivo* models. It is minimally invasive to the chick embryo, and it is therefore a potential approach to refinement of animal experimentation (Moreno-Jimenez et al., 2016).

In the context of wound healing, the cell differentiation assay should also be a reasonable method of choice. However, it was not well represented in our dataset, with only 5% of studies investigating the ability of PnD to promote skin cell differentiation *in vitro*. The same is true for the cytotoxicity and viability assays, which would also be useful before the application of the PnD *in vivo* in preclinical and clinical studies.

In general, the PnD treatment showed favorable effects in terms of cutaneous wound healing since it promoted cell proliferation, migration, and differentiation of cells *in vitro* and showed no cytotoxic effects (Table 1, Table 2, Table 3, Table 4, Table 5). In this regard, the functional assays demonstrated considerable potency, since the results from the preclinical studies also show a promoting effect of PnD on cutaneous wound healing (Pichlsberger et al., 2021). Nevertheless, evaluating the potency of the *in vitro* functional assays turned out to be delicate as the PnD tested *in vitro* were often not the same as those applied *in vivo*. For example, in the same study the PnD-derived CM was functionally tested *in vitro*, but whole perinatal cells were applied to the animal wound (Kim et al., 2012a; Edwards et al., 2014; Jin et al., 2016). For the development of more effective PnD therapies, this will need to be considered in future studies.

**TABLE 2:** Functional tests on perinatal cell-conditioned medium (CM) alone or compared to/or combined with perinatal cells.

**Perinatal cell-conditioned medium (CM)**			
**PnD**	**Functional *in vitro* tests**	**Outcome**	**Reference**
hAEC-CM	scratch wound assay	hAEC-CM substantially accelerated the migration of HDF.	(Jin et al., 2016)
hPMSC-CM	(i) Cell proliferation assay	Hypoxic hPMSC-CM inhibited the (i) proliferation and (ii) migration of HDF compared to normal medium and normoxic CM.	(Du et al., 2016)
	(ii) Scratch wound assay		
hUC-MSC-CM	(i) Cell cycle analysis	(i) hUC-MSC-CM caused a G0/G1-phase cell cycle arrest of HUVEC. (ii) HUVEC treated with hUC-MSC-CM had a significantly down-regulated expression of genes for IFN, TNF, IL-1, and IL-6, while the key genes involved with angiogenesis (VEGF, EGF, bFGF, and KDR) were up-regulated. (iii) hUC-MSC-CM significantly increased the proliferation of HUVEC.	(Sun et al., 2019)
	(ii) Cell differentiation assay		
	(iii) Cell proliferation assay		
	Cytotoxicity assay	50 and 100% (*V*/V) concentrations of the hUC-MSC-CM had a cytotoxic effect on HDF, contrary to 25% CM.	(Sabzevari et al., 2020)
hAMSC-CM	(i) Scratch wound assay	(i) hAMSC-CM significantly increased the migration rate of HDF and HUVEC. (ii) HUVEC treated with hAMSC-CM formed significantly longer tubes compared to untreated controls.	(Kim et al., 2012b)
	(ii) Tube formation test		

Perinatal cell-conditioned medium (CM) compared to / or combined with perinatal cells			
PnD	Functional in vitro tests	Outcome	Reference
a) hAMSC	(i) Cell apoptosis assay	hAMSC and hAMSC-CM (i) inhibited heat stress-induced apoptosis in HaCAT cells and HDF through activation of PI3K/AKT signaling and (ii) promoted their proliferation by activating GSK3β/β-catenin signaling. (iii) Higher HaCAT and HDF cell migration was observed in the presence of hAMSC and hAMSC-CM as compared to the control medium. (iv) hAMSC did not display tumorigenicity *in vitro*. (v) hAMSC-CM promoted HUVEC tube formation.	(Li et al., 2019)
b) hAMSC-CM	(ii) Cell proliferation assay		
	(iii) Scratch wound assay		
	(iv) Soft agar tumorigenicity test		
	(v) Tube formation assay		
a) hUC-MSC	(i) Chemotaxis assays	(i,ii, iv) Raw264.7 macrophages activated by hUC-MSC-CM enhanced chemotaxis, migration and angiogenesis of HUVEC with diabetic dysfunction. (iii) hUC-MSC and hUC-MSC-CM are capable of switching macrophages from the M1 phenotype to the M2 phenotype. The action of hUC-MSC-CM on macrophages or endothelial cells was inhibited by neutralizing antibodies against PGE2 or by the inhibition of PGE2 secretion from hUC-MSC.	(Zhang et al., 2020)
b) hUC-MSC-CM	(ii) Scratch wound assay		
	(iii) Transwell co-culture assay		
	(iv) Tube formation assay		
	(i) Cell metabolic activity assay	(i, ii) hUC-MSC seeded on scaffolds maintained higher metabolic activity than adipose tissue MSC during 48 h serum starvation. (iii, iv) hUC-MSC-CM promoted angiogenesis.	(Edwards et al., 2014)
	(ii) Cell adhesion on a scaffold		
	(iii) Tube formation test		
	(iv) CAM assay		
a) hAEC-CM	(i) Cell cycle analysis	(ii) hAEC-CM accelerated the migration and proliferation of keratinocytes and induced increased activity of phospho-ERK, phospho-JNK, and phospho-AKT. The blockade of phospho-AKT inhibited migration induced by hAEC-CM. (i, iii) Coculturing of keratinocytes with hAEC promoted keratinocyte proliferation by up-regulation of Cyclin D1, Cyclin D3 and Mdm2.	(Zhao et al., 2016)
b) hAEC	(ii) Scratch wound assay		
	(iii) Transwell co-culture assay		

Abbreviations: bFGF: basic fibroblast growth factor, CAM: chick chorio allantoic membrane, CM: conditioned medium derived from hAEC, hAMSC, hPMSC, hUC-MSC, hUC-MSC-End, EGF: epidermal growth factor, HaCAT: immortalized human keratinocytes, hAEC: human amniotic membrane epithelial cells, hAMSC: human amniotic membrane mesenchymal stromal cells, HDF: human dermal fibroblasts hPMSC: human placenta mesenchymal stromal cells, hUC-MSC: human umbilical cord mesenchymal stromal cells, hU-MSC-End: human umbilical cord mesenchymal stromal cells-endothelial transdifferentiated, HUVEC: human umbilical cord vein endothelial cells, IL-1, IL-6: interleukin-1, -6, KDR: kinase insert domain receptor, PGE2: prostaglandin E2, TNF: tumor necrosis factor, VEGF: vascular endothelial cell growth factor. Perinatal cell-conditioned medium (CM).

**TABLE 3:** Functional tests on Perinatal cell-derived small extracellular vesicles (sEV) alone or compared to perinatal cell-conditioned medium (CM).

**Perinatal cell-derived small extracellular vesicles (sEV)**			
**PnD**	**Functional *in vitro* tests**	**Outcome**	**Reference**
hUC-MSC-sEV	(i) Cell differentiation assay	(i) hUC-MSC-sEV inhibited α-SMA and collagen I and III expression in HDF cultivated at high cell density. (ii) hUC-MSC-sEV restrict HaCaT and HDF proliferation at high cell densities, but promote cell proliferation at low densities.	(Zhang et al., 2016)
	(ii) Cell proliferation assay		
	(i) Cell proliferation assay	hUC-MSC-sEV promoted the (i, ii) proliferation, (iii, v) migration, and (iv) tube formation of a HUVEC-derived cell line in a dose-dependent manner.	(Zhang et al., 2015)
	(ii) Cytotoxicity assay	
	(iii) Scratch assay	
	(iv) Tube formation assay	
	(v) Chemotaxis assay	
	(i) Cell proliferation assay	hUC-MSC-sEV promoted (i) the proliferation, (ii) migration and (iii) tube-formation of HUVEC. hUC-MSC-sEV contained Ang-2, and treatment with hUC-MSC-sEV enhanced the expression of the Ang-2 in HUVEC through exosome-mediated Ang-2 transfer.	(Liu et al., 2021)
	(ii) Chemotaxis assay	
	(iii) Tube Formation Assay	
**Perinatal cell-derived small extracellular vesicles (sEV) compared to perinatal cell-CM**			
**PnD**	**Functional *in vitro* tests**	**Outcome**	**Reference**
a) hUC-MSC-sEV	(i) Cell apoptosis assay	hUC-MSC-CM and hUC-MSC-sEV (i) decreased H_2_O_2_-induced cell apoptosis of HaCaT by inhibiting AIF and upregulating PARP-1 and poly ADP-ribose, (ii) increased HaCaT proliferation, in contrast to hUC-MSC-sEV-dp. (iii) hUC-MSC-CM improved the viability of HaCaT. (iv, vi) hUC-MSC-sEV and hUC-MSC-CM promoted cell migration relative to the hUC-MSC-sEV-dp. (vi) (v) ROS intensity in the hUC-MSC-sEV group and hUC-MSC-CM group was lower than in the control group.	(Zhao et al., 2020)
b) hUC-MSC-CM	(ii) Cell proliferation assay		
	(iii) Cell viability assay		
	(iv) Chemotaxis assay		
	(v) ROS generation assay		
	(vi) Scratch wound assay		
	(i) Cell proliferation assay	(i) hUC-MSC-sEV/Pluronic F-127 hydrogel promoted HUVEC proliferation better than hUC-MSC-sEV and hUC-MSC-CM. (ii) hUC-MSC-sEV and CM groups showed greater cell migration than the Pluronic F-127 hydrogel and control group. The hUC-MSC-sEV/Pluronic F-127 hydrogel group exhibited the best performance.	(Yang et al., 2020)
	(ii) Scratch wound assay		
a) hDMSC-sEV	Cell cycle assay Cell differentiation assay Cell proliferation assay Scratch wound assay Senescence assay ROS generation assay	(i) hDMSC-sEV enhanced proliferation of HG aged HDF, (iv) increased their migration rate, (ii) promoted differentiation of HG-aged HDF into myofibroblasts (increased α-SMA and collagen I protein expression), (v) inhibited senescence associated β-galactosidase expression and (vi) inhibited ROS generation in HG aged HDF. hDMSC-CM improved (iii) proliferation and (iv) migration of HDF. The sEV blocker GW4869 reduced both effects, indicating that hDMSC-sEV in hDMSC-CM probably enhance the proliferation and migration abilities of HDF.	(Bian et al., 2020)
b) hDMSC-CM			

Abbreviations; Ang-2: angiopoietin-2, AIF: apoptosis-inducing factor, α-SMA: alpha-smooth muscle actin, CM: conditioned medium derived from hDMSC, hUC-MSC, HaCAT: immortalized human keratinocytes, HDF: human dermal fibroblast, hDMSC: human decidua mesenchymal stromal cells, HG: high glucose, hUC-MSC: human umbilical cord mesenchymal stromal cells, PARP-1: poly ADP, ribose polymerase 1, ROS: reactive oxygen species, sEV: small extracellular vesicles derived from hDMSC, hUC-MSC, sEV-dp: conditioned medium depleted from small extracellular vesicle. Perinatal cell-derived small extracellular vesicles (sEV).

**TABLE 4:** Functional tests on perinatal tissues.

**Perinatal tissues**			
**PnD**	**Functional *in vitro* tests**	**Outcome**	**Reference**
Dehydrated hAM/chorion (EpiFix^®^)	(i) Cell proliferation assay	(i) Dehydrated hAM/chorion extracts caused a dose-dependent increase in HDF proliferation. (ii) Dehydrated hAM/chorion tissue allografts promoted migration of hMSC. (iii) Growth factors such as EGF, bFGF and TGF-1 were able to elute from dehydrated hAM/chorion into the saline.	(Koob et al., 2013)
	(ii) Chemotaxis assay		
	(iii) ELISA assay		
Dehydrated hAM/chorion (EpiFix^®^)	(i) Cell proliferation assay	(i) Dehydrated hAM/chorion extract promoted HMVEC proliferation. (ii) Dehydrated hAM/chorion tissue recruited migration of HUVEC. (iii) Dehydrated hAM/chorion extract increased endogenous production of over 30 angiogenic factors by HMVEC, including GM-CSF, angiogenin, TGF-β3, and HB-EGF Heparin-binding EGF-like growth factor.	(Koob et al., 2014)
	(ii) Chemotaxis assay		
	(iii) ELISA assay		
hAM	Cytotoxicity assay	Decellularized hAM enhanced the viability of hUC-MSC seeded onto the epithelial surface of hAM.	(Hashemi et al., 2020)

Abbreviations: bFGF: basic fibroblast growth factor, EGF: epidermal growth factor, GM-CSF: granulocyte macrophage colony-stimulating factor, hAM: human amniotic membrane, HB-EGF: Heparin-binding EGF-like growth factor, hMSC: human mesenchymal stromal cells, HMVEC: human microvascular endothelial cells, hUC-MSC: human umbilical cord mesenchymal stromal cells, TGF-1: transforming growth factor 1, TGF-β3: transforming growth factor β3.

**TABLE 5:** Functional tests on perinatal tissue extracts alone or combined with conditioned medium (CM).

**Perinatal tissue extracts**			
**PnD**	**Functional *in vitro* tests**	**Outcome**	**Reference**
hAM extract	(i) Cell apoptosis assay	(i) Higher concentrations of hAM extract increased the percentage of apoptotic and necrotic HDF. (ii, iii) hAM extract promoted HDF proliferation and migration.	(Momeni et al., 2018)
	(ii) Cell proliferation assay		
	(iii) Scratch wound assay		
Placental laminin	(i) Cell differentiation assay	(i) Placental laminin purified from hP extract promoted neuronal differentiation of neuronal cell line PC12 (ii) Non-toxic concentration of placental laminin for PC12 cell treatment was determined (0.17 lg/ml). (iii) Placental laminin accelerated migration and motility of mouse embryonic fibroblasts. (iv) Blocking of integrin receptor retarded neurite outgrowth in laminin treated PC12 cells.	(Mukherjee et al., 2020)
	(ii) Cell viability assay	
	(iii) Scratch wound assay	
	(iv) Receptor antagonist assay	
a) hAM powder	(i) Biocompatibility assay	(i) Heparinized human blood biocompatibility assay showed intact blood cells upon incubation with hAM powder or hAM powder + AV gel. (ii, iii) Media containing hAM powder + AV gel promoted HaCaT and HDF cell attachment and proliferation. (iv) hAM powder + AV significantly accelerated migration of HaCaT.	(Rahman et al., 2019)
b) hAM powder + AV	(ii) Cell attachment assay	
	(iii) Cell proliferation assay	
	(iv) Scratch wound assay	
Solubilized hAM	(i) Cell proliferation assay	(i) hAM-hyaluronic acid hydrogel accelerated proliferation of HDF and keratinocytes compared to controls. (ii) Keratinocytes and HDF remained viable following hAM-hyaluronic acid hydrogel encapsulation.	(Murphy et al., 2017)
	(ii) Cell viability assay		
hWJ-ECM	(i) Cell proliferation assay	(i) The HDF cell line HSF-PI 18 attached to, infiltrated into and proliferated on hWJ-ECM scaffolds. (ii) hWJ-ECM was not cytotoxic.	(Beiki et al., 2017)
	(ii) Cytotoxicity assay	
hWJ-ECM	(i) Cell differentiation assay	(i) hWJ-ECM promoted differentiation of HDF into myofibroblasts (confirmed by upregulation of α-SMA expression). (ii) hWJ-ECM treatment did not affect cell proliferation or (iii) cell viability of HDF. (iv) hWJ-ECM enhanced HDF migration.	(Bakhtyar et al., 2017)
	(ii) Cell proliferation assay	
	(iii) Cell viability assay	
	(iv) Scratch wound assay	

Perinatal tissue extracts combined with conditioned medium			
PnD	Functional *in vitro* tests	Outcome	Reference
a) hP–ECM	(i) Cell apoptosis assay	(i) hP-ECM-silk fibroin scaffolds-CM had no detrimental effect on hEK, (ii) provided a non-cytotoxic environment for HDF, hEK and hAMSC to adhere, infiltrate and proliferate, (iii) and promoted vascularization. (iv) Scaffold-CM promoted the migration of HDF and hEK.	(Rameshbabu et al., 2018)
b) hP-ECM-CM	(ii) Cytotoxicity assay	
	(iii) CAM Assay	
	(iv) Scratch wound assay	

**Abbreviations: α-SMA**: alpha-smooth muscle actin, **AV**: aloe vera, **HaCaT**: immortalized human keratinocytes, **hAM**: human amniotic membrane, **hAMSC:** human amniotic mesenchymal stromal cells, **HDF:** human dermal fibroblasts, **hEK**: human epidermal keratinocytes, **hP**: human placenta, **hP-ECM**: human placenta extracellular matrix, **hUC-WJ-ECM**: human umbilical cord Wharton´s jelly extracellular matrix.

### 2.4 Oral wound healing versus cutaneous wound healing

Special PnD, i.e. hAM, chorion and human Amnio-Chorionic Membrane (hACM) have been widely used for wound healing in oral reconstruction (Fenelon et al., 2018; Gulameabasse et al., 2020). For instance, hAM as a scaffold enhanced re-epithelialization of the oral cavity and reduced the contracture effects in moderate-sized defects (Lawson, 1985). The hAM has also been proven as useful biodegradable graft material for clinical vestibuloplasty. One week after the surgical intervention, epithelium started to migrate over the graft area from the margins, and the underlying connective tissue showed the formation of granulation tissue. The hAM completely degenerated after 3 weeks. Three months post intervention, epithelial tissue was restored and completely covered the graft area (Samandari et al., 2004). Measurement of blood flow to grafts used in vestibuloplasty revealed an increased blood flow to hAM grafts, whereas palatal grafts had a reduced blood flow during the same time period. Thus, angiogenesis was induced by hAM within 10–15 days, and the blood flow returned to normal by 30 days after surgery (Guler et al., 1997). These favorable effects of the hAM may be due to growth factors present in the amnion, such as basic Fibroblast Growth Factor (bFGF), Epidermal Growth Factor (EGF), TGFβ and IL-1 (Dadkhah Tehrani et al., 2020).

A corresponding animal study revealed that hAM transplanted on rabbit’s gingival wound accelerated the formation of granulation tissue at day 10 by significantly increasing the number of both fibroblasts and blood vessels. Thus, hAM induced rapid epithelialization and both granulation tissue and collagen formation, and suppressed inflammation i. e., the migration of polymorphonuclear cells at the wounded gingival site (Rinastiti et al., 2006).

In oral wound healing, the anti-adhesive properties of hAM in contact with healthy tissue are useful to prevent tissue adhesion in surgical procedures. The hAM transplant may function as an anatomical barrier towards fibrous tissue proliferation (Samandari et al., 2004; Fenelon et al., 2018), but further models are needed to improve understanding of this mechanism of action.

An interesting aspect in the context of wound healing is that oral wounds mainly heal without scarring. The keratinized *epidermis* of the skin shares some similarities with the oral mucosa, such as the stratified epithelium. In addition, also certain regions of the oral mucosa (gingiva, palate) show signs of keratinization, while the nonkeratinized floor of the mouth and buccal regions do not produce a stratum corneum (Wertz, 2021).

The main difference between skin and the keratinized regions of the oral mucosa is the relatively dry cutaneous surface, which is covered by the secretion products of sweat glands. Hair follicles can be found in most cutaneous regions except the palms and foot soles, and the apical surface is additionally covered by sebum. In contrast, the oral mucosa is constantly moist and well protected by the continuous secretion of mucous glands. The humid milieu may be one of the reasons that the healing of oral mucosal wounds is faster with minimal to no scar formation. There is a smaller inflammatory response with less neutrophils, macrophages, and T-cell infiltration, and the proliferation and migration rate of keratinocytes from oral mucosa is much more rapid as compared to skin keratinocytes (Turabelidze et al., 2014).

During the healing period of adult skin wounds rapid angiogenesis occurs leading to many more capillaries than in normal tissue. Compared to that, healing wounds of oral mucosa have a reduced angiogenic network but are composed of more mature vessels providing better oxygenation. As inflammatory cells produce a variety of proangiogenic factors, it can be assumed that the selective reduction of inflammation and angiogenesis may help to prevent excessive scarring (Dipietro, 2016).

Covering wounds with various types of dressings may help to keep the wound area in a humid milieu that allows controlled regeneration processes without excessive scarring. Most current *in vitro* assays are performed under fluid conditions, where the cells are exposed to culture medium. Functional assays on cell cultures using air-liquid-interface (ALI) would be helpful to mimic physiologic conditions even more closely.

## 3 Summary and conclusion

**FIGURE 2:**
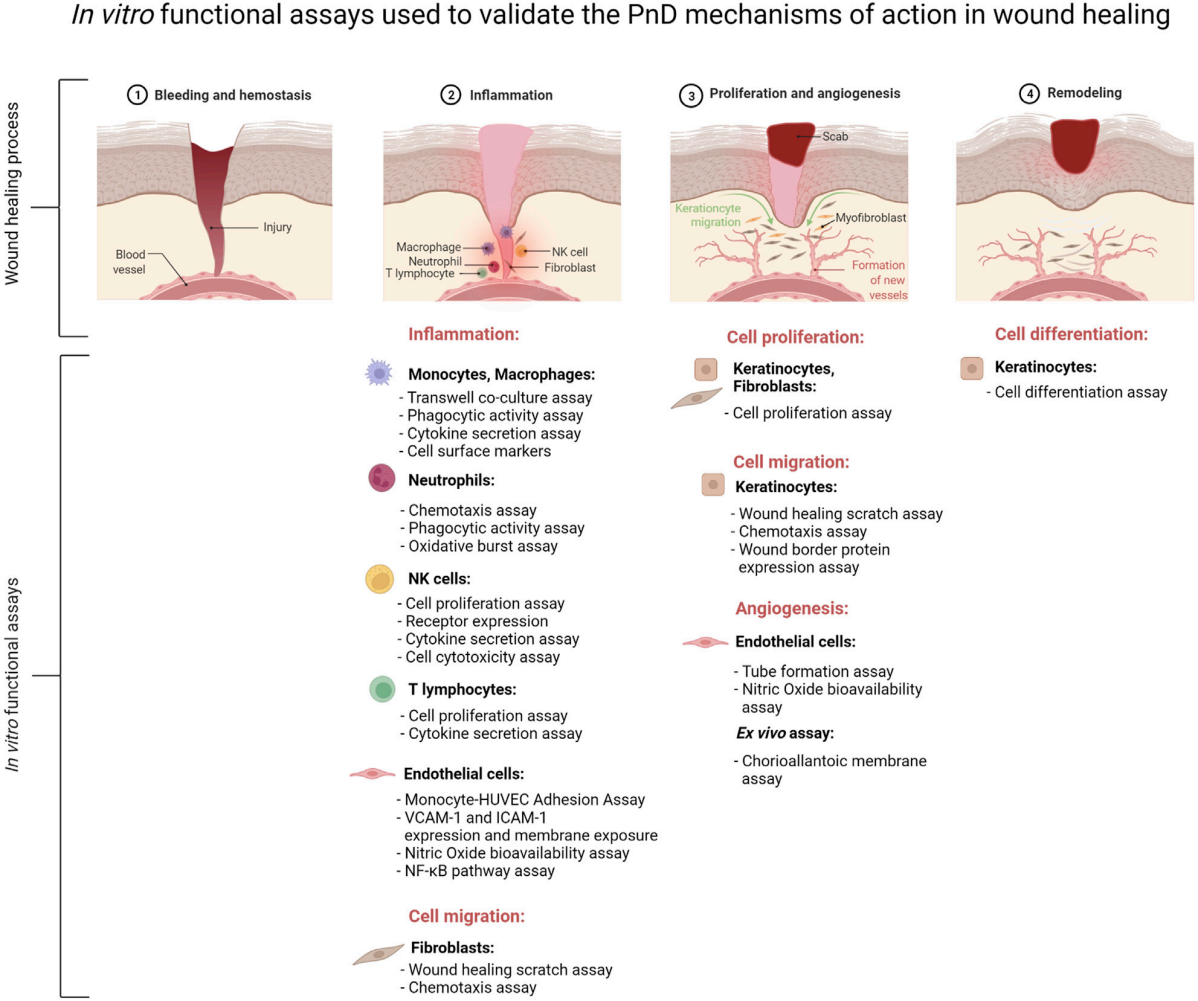
Schematic presentation of the major *in vitro* functional assays performed to validate the mechanisms of action of perinatal derivatives (PnD) in the context of inflammation, angiogenesis and remodeling during the complex process of wound healing. The figure was created using Biorender.com.

In this review, we addressed studies in which PnD were functionally tested *in vitro* before being applied to pre-clinical or clinical care with respect to inflammation, angiogenesis and wound healing (Figure 2). We have focussed on the types and combinations of PnD and the functional assays used as well as on the outcomes of the *in vitro* assays.

Inflammation is a complex physiological mechanism involving different types of immune cells, protein mediators, and metabolites, making it difficult to study the role of PnD in inflammation *in vitro*. Our analysis indicates that most of the available assays to determine the ability of PnD to control inflammation study the interaction of different types of PnD with a single type of immune cells. These *in vitro* studies are mainly based on transwell co-cultures of PnD with immune cells, or treatment of immune cells with PnD CM, allowing to differentiate between cell-to-cell contact or paracrine signaling, respectively. The following functional assays are the most frequently used for evaluating immune cell activity: cell proliferation assay, cytokine secretion assay, surface markers expression indicative of acquisition of an anti-inflammatory phenotype, cytotoxicity and phagocytosis assay. Oxidative burst capacity is another assay that has been used to validate the anti-inflammatory role of PnD over neutrophils. All specialized phagocytes i.e., neutrophils and macrophages, have the capacity to generate the respiratory burst (Dahlgren et al., 2007), and this assay could be included as an *in vitro* functional test of the anti-inflammatory capacity of PnD over these other phagocytic cells. Moreover, the role of PnD on vascular inflammation can be assessed by means of several functional assays using HUVEC as a valuable *in vitro* model of human vasculature (Pipino et al., 2022).

Among the different functional assays available, the following four assay types are considered as the gold standard for assessing wound healing *in vitro*: the cell proliferation, the scratch wound, the chemotaxis, and the angiogenesis assay. Of note, *in vitro* models with characteristics closer to those cells in chronic wounds are needed to attain all the benefits of the application of PnD to non-healing ulcers (Liarte et al., 2020b). Indeed, while chronic wound fluids are known to be rich in proinflammatory cytokines, such as the anti-proliferative TGFß, it has been shown that AM can inhibit TGFß signaling, restoring keratinocyte proliferation and migration (Ruiz-Canada et al., 2018; Ruiz-Canada et al., 2021). Recently, HaCaT cells had been shown to represent a phenotype better resembling the state of keratinocytes in chronic wounds (Liarte et al., 2020a).

Inflammation, angiogenesis, and wound healing are thoroughly regulated processes that play an important role in tissue regeneration. If the balance of these regulatory mechanisms is disturbed, excessive inflammatory reactions, misguided angiogenesis, delayed wound healing or excessive scarring can occur. A promising therapeutic option is treatment with PnD. To understand the mode of action of PnD, various functional assays are carried out. Furthermore, there is a certain risk that the PnD sample may be of varying quality due to donor variability. Thus, functional assays are also important for checking the quality of the PnD compound prior to preclinical and clinical use.

As the gap between cell culture experiments and a complete organism is difficult to bridge, animal models are needed to resolve questions about toxicology or pharmacology. We strongly suggest that functional *in vitro* testing of PnD is routinely performed before their application to animal models and patients. This helps to minimize animal numbers in animal experimentation in line with the Replacement, Reduction, and Refinement (3R) principles for more ethical use of animals in research (Strech and Dirnagl, 2019) and to ensure the safety and efficacy of the PnD applied to patients.

The present review is in line with the aims of the COST SPRINT Action (CA17116) (Silini et al., 2020) and will contribute to the establishment of guidelines for methods applied to cells and tissues to enable scientifically sound and reproducible research data to promote PnD into clinics.
